# Supplementary material for: Association between Clinical and Histopathological Findings in Intestinal Neuronal Dysplasia Type B: An Advance towards Its Definition as a Disease
Source: Life (Basel). 2023 May 12;13(5):1175. doi: 10.3390/life13051175 (PMC10221867; doi:10.3390/life13051175)
Supplement: Supplementary file 1 [file life-13-01175-s001.zip › life-2301087-supplementary.pdf]

Table S1: exploratory factor analysis applying the principal components method.

Variables	**Factor 1** (28% of the variation explained)	**Factor 2** (17% of the variation explained)	**Commonalities**
Sex	-0.36976	-0.11383	0.14968337
Born to term	0.14257	0.59061	0.36914302
Birth weight	0.32672	-0.39439	0.26228682
Problems in the neonatal period	0.63274	0.30084	0.49086572
Other malformations	0.24566	0.16446	0.08739573
Delayed meconium passage	0.25442	0.06318	0.06872026
Age of onset of symptoms	-0.29863	0.14387	0.10988133
Maximum number of days without bowel movement	0.04793	-0.75333	0.56980449
Number of bowel movements per week	0.00138	0.04238	0.00179796
Age at diagnosis	-0.70306	0.43236	0.68122894
Need for bowel washouts	0.55462	-0.2281	0.35964037
Rome IV Q1: < 2 bowel movements per week	0.59909	-0.3154	0.45838443
Rome IV Q2: > 1 episode of fecal incontinence per week	-0.24887	0.6455	0.47860546
Rome IV Q3: history of episodes of fecal retention	0.27569	0.92252	0.92704185
Rome IV Q4: painful or hard bowel movements	0.27569	0.92252	0.92704185
Rome IV Q5: large fecal mass present in the rectum	0.93576	0.24813	0.93721924
Rome IV Q6: large diameter stools that can obstruct the toilet	0.41256	0.17498	0.20082856
Abdominal distension	0.27569	0.92252	0.92704185
Abdominal pain	0.27569	0.92252	0.92704185
Rectal bleeding	0.58657	0.33643	0.45725263
Intestinal Symptom Index (ISI)	0.59803	0.68232	0.82320183
Number of plexuses with more than 7 neurons	-0.55234	0.13644	0.32369045
Number of giant ganglia	0.81355	0.1761	0.69287359
Number of neurons in 25 ganglia	0.78385	0.46596	0.831539
Maximum number of neurons per ganglia	0.65466	0.26321	0.49785417
Maximum width of a plexus	-0.13106	-0.14222	0.03740139
Area of a neuron	-0.70673	0.33984	0.6149547
Mean of neurons per ganglia	0.78385	0.46596	0.831539
Bud-like nerve cell groups along nerve trunks	0.45286	-0.00031	0.20508568
Ganglia with signs of immaturity	0.38736	0.38166	0.29571201
Small neurons	0.11609	-0.29623	0.10122901
Anisomorphic neurons	-0.5993	0.07521	0.36481879
Calretinin IHC expression in nerve fibers in the lamina propria of the mucosa	-0.34273	-0.03628	0.11877902
Calretinin IHC expression in heterotopic neurons in the muscularis mucosa	-0.03404	-0.65429	0.42926056
Calretinin IHC expression in heterotopic neurons in the lamina propria	-0.4923	0.48742	0.47993282
